# Cost-effectiveness of pegfilgrastim versus filgrastim for prevention of chemotherapy-induced febrile neutropenia in patients with lymphoma: a systematic review

**DOI:** 10.1186/s12913-022-08933-z

**Published:** 2022-12-30

**Authors:** Girma Tekle Gebremariam, Atalay Mulu Fentie, Kebede Beyene, Beate Sander, Gebremedhin Beedemariam Gebretekle

**Affiliations:** 1 School of Pharmacy, Addis Ababa University, Zambia Street, P.O. box: 1176 Addis Ababa, Ethiopia; 2 Department of Pharmaceutical and Administrative Sciences, University of Health Sciences and Pharmacy in St. Louis, St. Louis, USA; 3 Institute of Health Policy, Management, and Evaluation, University of Toronto, 155 College Street, Toronto, Ontario M5T 3M6 Canada; 4 Toronto Health Economics and Technology Assessment (THETA) Collaborative, University Health Network, 200 Elizabeth Street, Toronto, Ontario M5G 2C4 Canada; 5 Institute for Clinical Evaluative Sciences, 2075 Bayview Ave, Toronto, Ontario M4N 3M5 Canada; 6 Public Health Ontario, 480 University Ave, Toronto, Ontario M5G 1V2 Canada; 7 Centre for Vaccine-Preventable Diseases, Dalla Lana School of Public Health, University of Toronto, Toronto, Canada

**Keywords:** Cost-effectiveness, Febrile neutropenia, Lymphoma, Prophylaxis, Pegfilgrastim, Filgrastim

## Abstract

**Background:**

Febrile neutropenia (FN) is a prevalent and potentially life-threatening complication in patients with lymphoma receiving myelosuppressive chemotherapy. Pegfilgrastim is more effective than filgrastim as prophylaxis for FN. However, its usage has been limited because of its higher cost. Pegfilgrastim’s value for money remains unclear.

**Objective:**

To systematically review the cost-effectiveness of pegfilgrastim compared to filgrastim as a primary or secondary prophylaxis for chemotherapy-induced FN among patients with lymphoma.

**Methods:**

A systematic literature search was conducted in PubMed, EMBASE, Cochrane Library databases, and Google Scholar. The most widely used economic evaluations (cost-effectiveness analysis, cost-utility analysis and cost-benefit analysis) were included in the review. Data extraction was guided by the Consolidated Health Economic Evaluation Reporting Standards checklist, and the quality of reviewed articles was assessed using the Joanna Briggs Institute (JBI) checklist. Cost-effectiveness data were rigorously summarized and synthesized narratively. Costs were adjusted to US$ 2020.

**Results:**

We identified eight economic evaluation studies (two cost-utility analyses, three cost-effectiveness analyses, and three studies reporting both cost-effectiveness and cost-utility analyses). Half of these studies were from Europe (*n* = 4), the other half were from Iran, USA, Canada, and Singapore. Six studies met > 80% of the JBI quality assessment criteria. Cost-effectiveness estimates in the majority (*n* = 6) of these studies were for Non-Hodgkin Lymphoma patients receiving myelosuppressive chemotherapy with high-risk of FN (> 20%). The studies considered a wide range of baseline FN risk (17–97.4%) and mortality rates (5.8–8.9%). Reported incremental cost-effectiveness ratios ranged from US$ 2199 to US$ 8,871,600 per quality-adjusted life-year (QALY) gained, dominant to US$ 44,358 per FN averted, and US$ 4261- US$ 7251 per life-years gained. The most influential parameters were medication and hospitalization costs, the relative risk of FN, and assumptions of mortality benefit.

**Conclusions:**

Most studies showed that pegfilgrastim is cost-effective compared to filgrastim as primary and secondary prophylaxis for chemotherapy-induced FN among patients with lymphoma at a cost-effectiveness threshold of US$ 50,000 per QALY gained. The findings could assist clinicians and healthcare decision-makers to make informed decisions regarding resource allocation for the management of chemotherapy-induced FN in settings similar to those studied.

**Supplementary Information:**

The online version contains supplementary material available at 10.1186/s12913-022-08933-z.

## Introduction

Febrile neutropenia (FN) is a prevalent and potentially life-threatening complication of chemotherapy that is associated with substantial morbidity, mortality, and healthcare cost [1, 2]. It is a manifestation of neutropenic infection and commonly results in suboptimal delivery of myelosuppressive anti-cancer drugs as well as treatment delays or dose reductions which may compromise chemotherapy treatment outcomes [3]. In patients with FN risk greater than 20%, most current treatment guidelines recommend using long-acting granulocyte colony-stimulating factors (G-CSFs) as primary prophylaxis of FN starting from the first cycle of chemotherapy [4–7].

G-CSFs are biological growth factors that promote proliferation, differentiation, and activation of neutrophils in the bone marrow [8]. The most commonly used recombinant G-CSFs are filgrastim and its PEGylated formulation, pegfilgrastim. The use of these agents as a preventive measure of FN has been associated with reduced hospitalization and severity of FN [7, 9]. They are frequently indicated to reduce the duration and incidence of FN in patients with non-myeloid malignancies receiving myelosuppressive chemotherapy [10]. Due to a longer half-life and slower elimination rate than filgrastim, pegfilgrastim requires less frequent dosing than filgrastim. While pegfilgrastim requires only single-dose chemotherapy per cycle, filgrastim is needed until neutrophil counts recover, with an average of 6–11 days per cycle [11]. Meta-analyses of comparative effectiveness studies suggested that pegfilgrastim has superior efficacy in reducing FN risk, FN-related mortality, and all-cause hospitalization [12–15]. Another meta-analysis of five trials demonstrated that pegfilgrastim had better efficacy than filgrastim with respect to FN risk reduction and shortening the duration of FN [16]. A systematic review of “real world” comparative effectiveness studies found that pegfilgrastim prophylaxis was associated with a decreased risk of FN and FN-related complications than filgrastim [17]. However, wider use of pegfilgrastim has been limited because of its higher purchasing cost in many countries [7].

Cost-effectiveness analyses (CEA) of the prophylactic use of pegfilgrastim and filgrastim for chemotherapy-induced FN have been reported for patients with Non-Hodgkin Lymphoma (NHL) who received cyclophosphamide, doxorubicin, vincristine, and prednisone with or without rituximab (R-CHOP) based chemotherapy [18–20]. Pegfilgrastim was found cost-effective compared to filgrastim in some of these investigations [21–23], but not in others [19, 24]. To our knowledge, this is the first comprehensive systematic review to assess the cost-effectiveness of pegfilgrastim versus filgrastim as a prophylactic strategy for chemotherapy-induced FN in patients with lymphoma. Owing to budgetary constraints and the need for value-based healthcare services, our systematic review could help to inform prescribing guidelines and policy decisions in resource allocation.

## Methods

### Literature search strategy

This systematic review was conducted in accordance with the Preferred Reporting Items for Systematic Reviews and Meta-analyses (PRISMA) [25], and the review protocol was registered in the International Prospective Register of Systematic Reviews (PROSPERO, ID = CRD42020220276). The search strategy was developed by the research team in consultation with a subject librarian and information specialist. We performed a systematic literature search in PubMed, EMBASE, Google Scholar, and the Cochrane Library (which includes the Health Technology Assessment Database, the National Health Service Economic Evaluation Database, and the Database of Abstracts of Reviews of Effects). Each database was scanned from inception up to November 2022 for full economic evaluations, and comparative analysis of alternative interventions in terms of both costs and consequences, i.e., health outcomes. The search strategy was adapted to each database. Keywords and medical subject headings for the database search were economic evaluation, cost-effectiveness analysis, cost-utility analysis, cost-benefit analysis, pharmacoeconomic evaluation, lymphoma, febrile neutropenia, G-CSF, filgrastim, and pegfilgrastim. The full search strategy is summarized in Supplementary file-1. In addition to database search, we cross-checked manually the references of all included studies.

### Eligibility criteria

Articles were included in this review if (i) study design and methods for economic evaluations were fully described; (ii) they were among the widely used economic evaluations (cost-effectiveness analysis, cost-utility analysis, and/or cost-benefit analysis); (iii) both costs and consequences were presented for the two interventions; iv) filgrastim and pegfilgrastim were used as primary or secondary prophylaxis for FN in lymphoma patients; and (v) patients histologically diagnosed with lymphoma. Abstract, case reports, commentaries, a letter to the editor, and unpublished reports were excluded. We excluded articles written in a non-English language. Other types of economic evaluations, such as cost-minimization analysis, were also excluded, as it is less commonly performed and only appropriate in rare circumstances., we also excluded economic evaluations that did not compare prophylactic use of pegfilgrastim versus filgrastim. We included reported outcomes related to net benefit or benefit to cost ratio, and an incremental cost per unit of health outcome, including cost per quality-adjusted life-year (QALY) gained, cost per FN averted, and cost per life years (LYs) gained.

### Data extraction and quality assessment

With the consensus of the research team, we created a standardized electronic data-charting form to collect data from eligible studies. Three authors (GTG, AMF and GBG) conducted pilot data extraction to refine the data extraction tools. Subsequently, title, abstract, and full article screening and quality appraisal were performed independently by two investigators (GTG and AMF). Disagreements were resolved by consensus or in consultation with other authors (GBG, BS, and KB).

The data extraction was guided by the Consolidated Health Economic Evaluation Reporting Standards (CHEERS) checklist [26]. Extracted study characteristics included author name, publication year, country, target population, type of prophylaxis, type of economic evalutions, study perspective, analytical approach (model type), time horizon, comparator, discount rate, year of valuation, study outcome measures (incremental cost-effectiveness ratio (ICER), FN averted, life years gained, mortality rates, medication cost, drug effectiveness), influential parameters, type of sensitivity analysis, and funding source. We assessed the methodological quality of each reviewed study using the Joanna Briggs Institute (JBI) checklist for economic evaluations [27]. Studies were considered as high quality if they met > 80% of the applicable JBI checklist criteria.

### Data analysis

We descriptively summarized the study characteristics. For comparability reasons, all ICERs were adjusted to US$ 2020 by using purchasing power parity (PPP) rates from the Organization for Economic Cooperation and Development (OECD) and US Department of Labour inflation rates [28, 29]. The adjusted ICER estimates were compared against two cost-effectiveness thresholds: US$ 50,000 per QALY gained and the World Health Organization recommended threshold of one times the country’s Gross Domestic Product per capita (1xGDP) per QALY gained [30, 31]. The GDP data were obtained from the World Bank [30]. We summarized the cost-effectiveness of primary and secondary prophylaxis that reported cost-effectiveness in cost per QALY gained, cost per FN averted, and cost per LYs gained. A meta-analysis of cost-effectiveness studies was not undertaken owing to the heterogeneity of the study settings, model type, parameters used, population, and study perspectives.

## Results

### Study characteristics

**Fig. 1:**
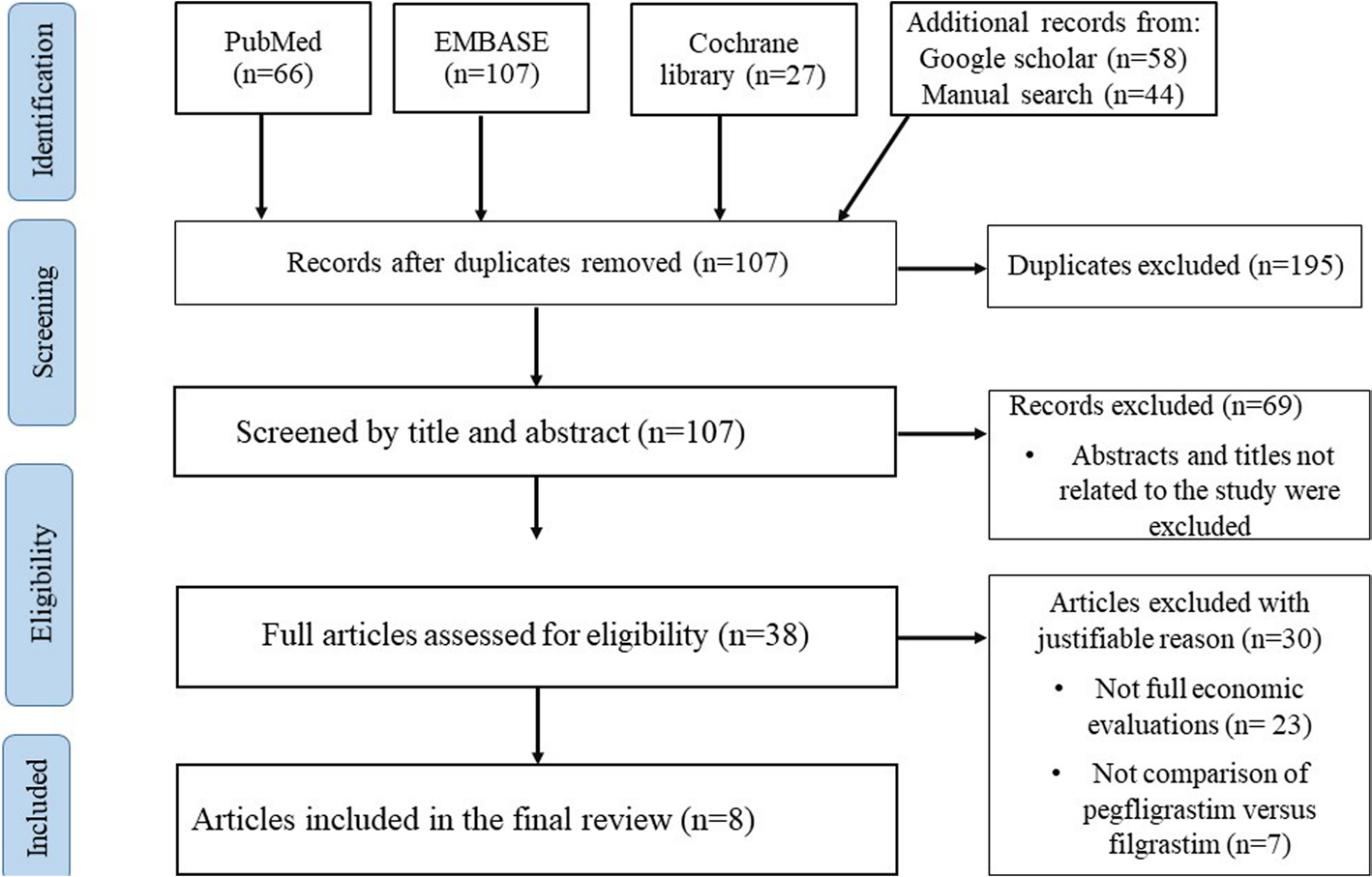
Preferred Reporting Items for Systematic Reviews and Meta-Analyses (PRISMA) flow diagram

Our search strategy identified 302 articles. Of these, 107 non-duplicate articles underwent title and abstract screening. The abstracts and titles of 69 studies were not related to our topic, and these studies were excluded at this stage. The full texts of the remaining 38 articles were thoroughly screened in detail, and 23 non-full economic evaluations and seven studies that did not compare the cost-effectiveness of pegfilgrastim and filgrastim were removed. Eight studies met the eligibility criteria and were included in the final review (Fig. 1). The studies were published between 2009 and 2017, where half of them were conducted in European countries [21, 23, 31, 32] and most of the studies (*n* = 7) were from high-income countries. Most studies (*n* = 6) were conducted among a hypothetical cohort of NHL patients aged greater than 18 years. Six studies were industry-sponsored [19, 21–23, 31, 32].

In five studies [19, 21–24] patients were on R-CHOP chemotherapy. The included studies reported an incremental cost per QALY gained (*n* = 2) [19, 21] and cost per FN averted (*n* = 3) [31–33]. The remaining studies (n = 3) [22–24] reported more than two outcome measures such as cost per QALY gained, cost per FN averted, and/or cost per LYs gained albeit over different time horizons. Of the eight reviewed studies, three were based on Markov models [19, 23, 24], two on decision trees [22, 33], and one on a mathematical model [21]. The remaining two studies were conducted alongside randomized clinical trials (RCTs) [31, 32] (Tables 1, 2).

**Table 1:** Study characteristics

Authors/year	Country	Perspective	Types of EE	Model type	Study population	Intervention^**a**^	Comparator	Type of prophylaxis
*Fust* et al. *2017* [23]	Belgium	Healthcare payer	CEA and CUA	Markov	205 elderly patients with aggressive NHL	Pegfilgrastim	6 and 11 days of filgrastim	Primary and secondary
*Ravangard* et al.*2017* [33]	Iran	Healthcare payer	CEA	Decision tree	131 patients with relapsed NHL, aged 19–72	Pegfilgrastim	1 and 3 days of filgrastim	Primary
*Wang* et al. *2016* [24]	Singapore	Hospital	CEA and CUA	Markov	Hypothetical cohort of 55-year-old patients with NHL	Pegfilgrastim	7 days of biosimilar filgrastim	Primary and secondary
*Lathia* et al. *2013* [19]	Canada	Healthcare payer	CUA	Markov	Hypothetical cohort of 64-year-old patients with DLBCL	Pegfilgrastim	10 days of filgrastim	Primary
*Perrier* et al.*2013* [32]	France	Hospital	CEA	Not specified	151 patients > 18 years with confirmed lymphoma	Pegfilgrastim	Average 6 days of filgrastim	Primary
*Sebban* et al.*2012* [31]	France	Hospital	CEA	Not specified	151 patients *>* 18 years with confirmed lymphoma	Pegfligrastim	Average 6 days of filgrastim	Primary
*Whyte* et al. *2011* [21]	UK	Healthcare payer	CUA	Mathematical	Hypothetical patients age of 63 years with aggressive NHL	Pegfilgrastim	6 and 11 days of filgrastim	Primary and secondary
*Lyman* et al. *2009* [22]	USA	Healthcare payer	CEA and CUA	Decision tree	Hypothetical cohort of 65-year-old patients with intermediate- or high-grade NHL	Pegfilgrastim	6 days of filgrastim	Primary

^a^Pegfilgrastim was given per cycle of chemotherapy

*FN* Febrile neutropenia, *EE* Economic Evaluation, *DLBCL* Diffuse large B-cell lymphomas, *CEA* cost-effectiveness analysis, *CUA *Cost-utility analysis, *NHS* National Health Service, *USA* United State of America, *UK* United Kingdom, *NHL* Non-Hodgkin lymphoma

**Table 2:** Study characteristics

Authors/year	Economic outcome measures	Time horizon	Sensitivity analysis	Influential parameters	Treatment	Discount rate (%)	Funding
*Fust* et al.*, 2017* [23]	Cost per QALY, cost per FN averted and cost per LYs	Lifetime	Deterministic and Probabilistic	RR of FN, mortality RR, RDI, and medication cost	R-CHOP	1.5	Amgen, Biotechnology company
*Ravangard* et al. *2017* [33]	Cost per FN averted	Not specified	Deterministic	Medication cost	ESHAP	NA	Not reported
*Wang* et al.*, 2016* [24]	Cost per QALY and cost per FN averted	18 weeks	Probabilistic Deterministic and TWSA	Medication cost, FN avoided	R- CHOP	NA	Not reported
*Lathia et a.l, 2013* [19]	Cost per QALY	18 weeks	Deterministic and Probabilistic	Hospitalization and medication cost	R-CHOP	NA	CIHR and Amgen Canada, Biotechnology company
*Perrier* et al.*2013* [32]	Cost per FN averted	100 days	Deterministic and Probabilistic	Length of hospital stay and Medication cost	Not specified	NA	Amgen, Biotechnology company
*Sebban* et al.*2012* [31]	Cost per FN averted	100 days	Deterministic and Probabilistic	Medication cost	Not specified	NA	Amgen, Biotechnology company
*Whyte* et al.*, 2011* [21]	Cost per QALY	Lifetime	Probabilistic	RR of FN, CET, RDI, age at dignosis and medication cost	R + CHOP	3.5	Amgen, Biotechnology company
*Lyman* et al.*, 2009* [22]	Cost per QALY, cost per LYs, cost per FN averted	Lifetime	Deterministic and Probabilistic	Medication cost, RR of FN, mortality RR, and baseline risk	CHOP	3	Amgen, Biotechnology company

*CET* Cost-effectiveness Threshold, *CHOP* ±*R* Cyclophosphamide, doxorubicin, vincristine, and prednisone plus/minus rituximab, *FN* Febrile neutropenia, *IHR* Canadian Health Research, *QALY* Quality adjusted life years, *LYs* Life year saved, *ESHAP* Etoposide, Methylprednisolone, cytarabine, cisplatin, *NA* Not Applicable, *TWSA* Two-way sensitivity analysis, *RDI* Relative dose intensity, *RR* Relative Risk

Base-case analyses were conducted from a healthcare payer perspective (*n* = 5) [19, 21–23, 33] and hospital perspective (*n* = 3) [25, 31, 32]. The studies modelled from 14 weeks to lifetime horizon. In three studies, discount rates for costs and health outcomes ranged from 1.5 to 3.5% [30, 33, 34], but five studies did not discount because the time horizon was less than 1 year [19, 24, 31–33]. All studies performed sensitivity analysis. Six studies reported both one-way and probabilistic sensitivity analyses [19, 21–23, 31, 32], whereas one study only reported probabilistic sensitivity analysis [24] and the other one reported one-way sensitivity analysis [33].

**Fig. 2:**
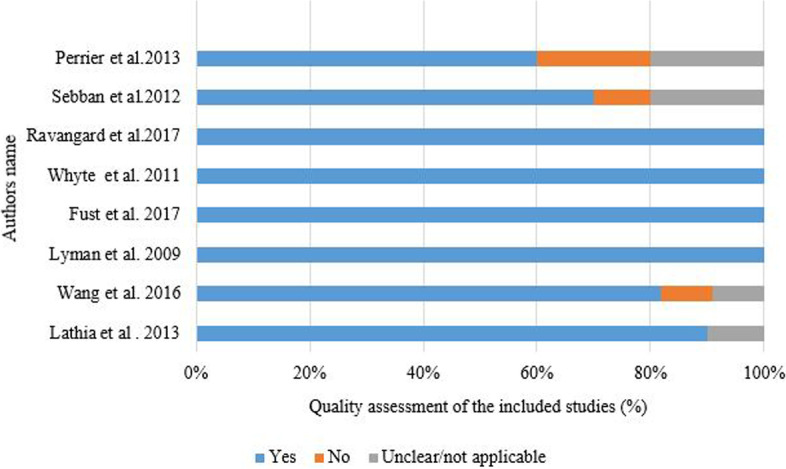
Percentage of quality appraisal results of included articles

Six studies met at least 80% of the JBI quality assessment criteria and were considered high quality [19, 21–24, 33] (Fig. 2). The remaining two studies were considered moderate quality [31, 32]. Most studies did not meet the criterion “Are costs and outcomes adjusted for differential timing?” in the JBI checklist for economic evaluations (Supplementary file-2).

### Study outcomes

#### Effectiveness measure

The reviewed studies used different effectiveness assumptions including mortality and survival benefits. Meta-analyses of RCTs of each G-CSF compared to no G-CSF prophylaxis are used in each study to examine the efficacy of the two G-CSFs in reducing FN risk. The studies reported that G-CSF administration increasing the likelihood that patients would receive the full planned chemotherapy dose (i.e., relative dose intensity (RDI) > 90%); reducing FN-related mortality and improving long-term survival [21, 22]. Consistent with the above findings, in Perrier et al*’s* [32] study absolute neutrophil count recovery was found to be more rapid for pegfilgrastim compared to filgrastim. The case fatality for hospitalized patients who were taking pegfilgrastim was estimated to be 5.8–8.9% compared to the baseline FN risk that ranged from 17 to 97.4%. According to Ravangard et al*’s* study [33], pegfilgrastim, 3 days and 1 day filgrastim treatments avoided 0.97, 0.95, and 0.83% of FN cases, respectively. In contrast, two studies assumed that G-CSF had no effect in reducing mortality [19, 24]. A summary of clinical parameters is presented in Table 3.

**Table 3:** Summary of cost and clinical parameters

Authors/ year	Cost of G-CSF (per cycle)	FN hospitalization cost per day	FN baseline risk (%)	FN mortality risk (%)	FN RR of pegfilgrastim^**a**^	Survival benefit of pegfilgrastim	Source of efficacy
*Fust* et al. *2017* [23]	PP of filgrastim = £ 18,170 (6 days) and £8862 (11 days), pegfligrastim = £ 19,149	US$ 7183	21	5.8	0.66 (11 days) 0.41 (6 days)	Yes	Meta-analysis
*Ravangard* et al.*2017* [33]	Pegfilgrastim = US$ 5299, filgrastim = US$ 4959 (3 days), filgrastim = US$ 5808 per dose	Not specified	Not specified	Not specified	Not specified	Not specified	Primary data
*Wang* et al. *2016* [24]	Pegfilgrastim = US$ 532 per dose, filgrastim =300 μg per dose = US$ 352 (7 days)	Pegfilgrastim = US$ 22,135 filgrastim = US$ 9588	41.8	NR	0.89	No	RCT
*Lathia* et al. *2013* [19]	Pegfilgrastim 6 mg = CAN$ 2422, filgrastim 300 μg per dose = CAN$ 1740 (10 days)	CAN$ 1012	64	NR	0.58	No	Meta-analysis
*Perrier* et al.*2013* [32]	Pegfilgrastim = US$ 25,024, filgrastim = US$ 28,700		Not specified	Not specified	Not specified	Not specified	RCT
*Sebban* et al.*2012* [31]	Pegfilgrastim = US$ 23,256, filgrastim = US$ 25,448	Pegfilgrastim = US$ 20,725,filgrastim = US$ 22,236	97.4	Not specified	Not specified	Not specified	RCT
*Whyte* et al. *2011* [21]	Filgrastim = £ 470 (6 days) and £ 862 (11 days), pegfligrastim = £ 686	£ 235	17 for age 63 years 45 at age 72 years	8.9	0.53	Yes	Systematic review
*Lyman* et al. *2009* [22]	Pegfilgrastim = US$ 2142, filgrastim = US$ 1596 (6 days)	US$ 15,921	27.9	5.8	0.52	Yes	Literature review

^a^*RR* Relative risk ratio, *FN* Febrile Neutropenia, *PP* Primary prophylaxis, *CAN* Canadian Dollar, *NR* Not Reported, *RCT* Randomized clinical trial

#### Cost measure

The estimation of costs varied in the studies, with an incremental cost of pegfilgrastim compared to filgrastim ranging from US$ 274 to US$ 6410. Since all studies were conducted from viewpoints of either the hospital (*n* = 3) or healthcare payer (*n* = 5), the costs considered in the analyses were primarily direct medical costs (such as cost of treatment, hospitalization, physician visit, laboratory, and imaging). In the majority of the studies [19, 22–24], costs were obtained from public health sources or government databases, but in the UK study conducted by Whyte et al. [21], list market price of medication was taken in the analysis. In Fust *et. al.* [23] study, costs estimate related to hospitalization for FN treatment were taken from literature study. Medication administration cost was not included in the models of two studies because patients self-administered the medication [23, 24]. The costs of chemotherapy for the patients in both pegfilgrastim and filgrastim arms of all the studies were the same. In two studies, costs data were collected alongside RCT from the hospital’s point of view [31, 32]. A summary of cost parameters including costs of G-CSF per cycle, FN hospitalization cost per day, and the incremental cost are presented in Tables 3 and 4.

**Table 4:** Summary of the cost-effectiveness of pegfilgrastim versus filgrastim in patients with lymphoma

Authors, y	Valuation year (currency measure used)	Incremental cost (US$2020)	Incremental effects	Original ICER	Adjusted ICER (US$2020per unit health outcome)	1xGDP (US$ 2020)	Authors conclusion and recommendation
*Fust* et al. *2017* [23]	2014 (€)	US$ 1540 (6 days) US$ 452 (11 days)	0.374 FN averted, 0.303 LYs gained and 0.268 QALY gained (6 days) 0.118 FN averted, 0.106 LYs, and 0.094 QALY (11 days)	US$ 3653 per QALY US$ 2617 per FN averted, US$ 3231 per LYs	US$ 5749 per QALY US$ 4120 per FN averted; US$ 5085 per LYs gained, US$ 3828 per FN averted US$ 4261 per LYs, US$ 4805 per QALY	44,594	Pegfilgrastim appears cost effective compared to other prophylaxis strategies
*Ravangard* et al.*2017* [33]	2014 (US$)	US$ 378 and US$ 566	0.02 and 0.14 FN averted	US$ 17,000 per FN averted (dominant) and - US$ 3635.7 (dominant)	US$ 18,89 per FN averted and US$ 404 per FN averted	2423	Pegfilgrastim strategies were more cost effective
*Wang* et al. *2016* [24]	2013 (US$)	US$ 274 at cycle 1 and 2, US$ 887 at all cycles	0.0001 QALY; 0.01 FN prevented (at cycle 1 and 2), 0.02 FN prevented (at all cycles)	US$ 4,058,623 QALY gained at all cycles and US$ 24,300 per FN prevented (cycle 1) US$ 39,300 per FN prevented (cycle 2)	US$ 8,871,600 per QALY gained, US$ 27,428 per FN averted, US$ 44,358 per FN averted	59,798	Pegfilgrastim is cost effective
*Lathia* et al. *2013* [19]	2012 (CAN$)	US$ 2228	0.0009 QALY	CAN$ 2,611,000 per QALY	US$ 2,475,344 per QALY gained	43,258	Dominated, pegfligrastim is not cost effective
*Perrier* et al.*2013* [32]	2009 (€)	US$ 6410	0.22 FN avoided	Dominant	US$ 29,135 per FN averted	38,625	Pegfilgrastim dominate filgrastim
*Sebban* et al.*2012* [31]	2009 (€)	US$ 3822	0.22 FN avoided	Dominant	US$ 17,372.95 per FN averted	38,625	Pegfilgrastim was cost-effective
*Whyte* et al. *2011* [21]	2010 (€)	US$ 345 (6 days), US$ 6397 (11 days)	0.056 QALY	US$ 3625 per QALY and dominant	US$ 6159 per QALY US$ 14,229per QALY gained	40,285	Pegfilgrastim was the most cost-effective of G-CSF
*Lyman* et al. *2009* [22]	2006 (US$)	US$ 341	Scenario 1 = 0.047 LYs, Scenario 2 = 0.042 QALY,0.174 LYs, Scenario 3 = 0.155 QALY	US$ 6190 per QALY, US$ 2167 per FN avoided, US$ 5532 per LYs	US$ 8114 per QALY US$ 2199 per QALY US$ 2840 per FN averted US$ 7251 per LYs	63,544	Pegfilgrastim is cost-effective compared to 6 days filgrastim

*CET* Cost-effectiveness Threshold, *GDP* Gross Domestic Product, *y* Year of publication

To convert the monetary unit to US$ 2020, first, the buying power of each country’s currency is changed to US$ by using purchasing power parity (PPP) rates from the Organization for Economic Cooperation and Development (OECD). Then, the United States Bureau of Labor Statistics consumer price index was used to inflate to US$ 2020 value from different years [28, 29]

#### Cost-effectiveness reported in cost per QALY

Accordingly, five studies reported ICER in cost per QALY with ICER estimates ranging from US$ 2199 to US$ 2,475,344 per QALY gained from the healthcare perspective and US$ 8,871,600 per QALY from the hospital perspective [19, 21–24]. Four studies reported ICERs below the cost-effectiveness threshold of US$ 50,000 per QALY and 1xGDP per capita per QALY [19, 21, 22, 24]. In these studies [19, 21, 22, 24], cost-effectiveness of primary prophylaxis of pegfilgrastim versus filgrastim ranged from dominant to US$ 14,229 per QALY. In a study conducted by Whyte et al. (2011) [21], the ICERs of single-dose per cycle pegfilgrastim used as primary and secondary prophylaxis compared to 6 cycles of filgrastim were US$ 6159 per QALY and US$ 14,229 per QALY, respectively. The highest estimates of ICERs were reported from the healthcare payer perspective over a time horizon of 18 weeks in Canada (US$ 2,475,344 per QALY) [19]; and the hospital perspective in Singapore (US$ 8,871,600 per QALY) [24], respectively. On the other hand, three studies conducted from the healthcare perspective [21–23] reported ICER estimates from US$ 2199 to US$ 14,229 per QALY. A study conducted by Lyman et al. (2009) [22], based on the premise that pegfilgrastim reduces FN-related mortality and improved long-term survival (i.e., RDI > 90%), reported ICER estimate of US$ 2199 per QALY from the US payer perspective. A Singaporean study among a hypothetical cohort of 55-year-old patients with NHL demonstrated that pegfilgrastim use as primary prophylaxis of FN was cost-effective at cycles 1 and 2 compared to filgrastim, but it was not cost-effective over six cycles of chemotherapy (adjusted ICER of US$ 8,871,600 per QALY) [24]. Table 4 summarizes the incremental cost, benefit, and health outcomes of each study, as well as ICER estimates (original and adjusted).

#### Cost-effectiveness reported in cost per FN averted

There were 6 studies [22–24, 31–33] in which cost-effectiveness was reported in cost per FN averted, of which two were an extension of comparative effectiveness RCTs [31, 32]. The ICERs for pegfilgrastim primary prophylaxis ranged from dominant (i.e., pegfilgrastim being less costly and more effective) to US$ 44,358 per FN averted. Two studies [22, 32] evaluated the cost-effectiveness of pegfilgrastim primary prophylaxis compared to filgrastim (6 and 11 days) from a healthcare payer perspective over a lifetime time horizon. The reported ICERs ranged from US$ 2199 to US$ 4120 per FN averted. A Singaporean study from a hospital perspective reported adjusted ICER estimates of US$ 27,428 per FN averted, and US$ 44,358 per FN averted at cycles 1 and 2, and all cycles of chemotherapy regimen, respectively [24]. A study conducted by Ravangard et al. [33] and Sebban et al. [31] among relapsed NHL on Etoposide, Methylprednisolone, cytarabine, cisplatin (ESHAP) chemotherapy regimen reported the degrees of FN prevented by single-dose pegfilgrastim versus a single day and 3 days filgrastim prophylaxis strategy. In these studies, pegfilgrastim was dominant compared to single-dose filgrastim and it resulted in ICER of US$ 18,890 per FN averted compared with 3 days filgrastim [24].

#### Cost-effectiveness reported in life years

Two studies reported the cost-effectiveness of pegfilgrastim prophylaxis in cost per LYs gained [22, 23]. These studies were conducted from the payer’s perspective among patients with aggressive NHL and reported ICER estimate from US$ 4261- US$ 7251 per LYs. In a hypothetical cohort of 65-year-old patients with high-risk NHL, considering a survival benefit of pegfilgrastim in avoiding FN mortality, Lyman et al. [22] reported an ICER of US$ 7251 per LYs. Fust *et. al.* [23] compared single-dose pegfilgrastim prophylaxis per cycle with 6 and 11 days of filgrastim per cycle and reported adjusted ICER of US$ 5085 per LYs and US$ 4261 per LYs, respectively.

#### Influential parameters

Several variables have influenced the sensitivity of ICER estimates in the reviewed articles. The most influential parameters reported across studies were medication cost, relative risk of FN between pegfilgrastim and filgrastim, chemotherapy regimen RDI, FN case-fatality rate, hospitalization cost, and baseline FN risk. Other important input variables were whether the G-CSF affects mortality, progression-free survival, and disease-free survival benefit of the treatment.

## Discussion

Our systematic review identified eight relevant economic evaluation studies comparing the cost-effectiveness of pegfilgrastim compared to filgrastim as a primary or secondary prophylaxis strategy among lymphoma patients with baseline FN risk of more than 20%. Most of the studies showed that pegfilgrastim prophylaxis to be cost-effective for primary and secondary prophylaxis of chemotherapy-induced FN compared to filgrastim in patients with lymphoma [19, 21–24, 31–33]. In five cost-utility studies [19, 21–24], the ICER estimates varied from dominant to US$ 8,871,600 per QALY gained. The majority of ICER estimates fell far below the cost-effectiveness threshold of US$ 50,000 per QALY and 1xGDP per capita per QALY. For studies that measured health outcomes in natural units, they reported an ICER value from US$ 2840 to US$ 44,358 per FN avoided, and US$ 426 to US$ 7251 per LYs gained.

These wide ranges of ICER estimates reported could be attributed to the analytical perspective adopted, costing approaches, health utility weights used and settings. It is noteworthy that different countries have distinct healthcare systems, heterogeneous service delivery, and measure costs from different viewpoints. All the studies were conducted from either the healthcare payer or hospital perspective. The studies conducted from the hospital perspective reported pegfilgrastim to be cost-effective than those undertaken from the healthcare payer perspective. Filgrastim treatment requires more visits resulting in increased travel expenditures as well as additional caregiver or patient costs related to missed productivity. However, the reviewed articles did not consider these indirect costs. Had these costs been considered, pegfilgrastim primary and secondary prophylaxis would likely to be cost-effective compared to filgrastim than reported. We suggest future comprehensive economic evaluation be carried out from a societal perspective with consideration of indirect costs of prophylaxis.

Our study demonstrated the cost-effectiveness of pegfilgrastim primary and secondary prophylaxis. Pegfilgrastim prophylaxis was found to be cost-effective in the majority of the reviewed studies. The review showed that relative risk of FN and medications cost had the greatest sensitivity to changes in ICER estimates. The cost-effectiveness of primary prophylaxis with pegfilgrastim appeared to be primarily contingent on assumed survival benefits (i.e., reduced FN associated deaths, progression-free survival). The majority of reviewed studies assumed that pegfilgrastim has survival benefits. The only exception to this was the Canadian study [19] which assumed that pegfilgrastim does not improve the overall survival or progression-free survival of patients. This might explain the small incremental health gains (0.0009 QALY) associated with pegfilgrastim in this study. In a US study, the probability of pegfilgrastim primary prophylaxis being cost-effective compared with filgrastim became 50, 80, and 91% with a cost-effectiveness threshold of US$ 15,000 per QALY, US$ 30,000 per QALY, and US$ 50,000 per QALY, respectively. This shows that a significant variation in ICER estimate was owning to influential variables that change significantly the cost-effectiveness acceptability curve. Six out of eight reviewed studies were funded by a pegfilgrastim innovator pharmaceutical company, which could introduce bias and may favour the new agent. We recommend future independent studies to determine the cost-effectiveness of pegfilgrastim versus filgrastim.

We extracted all base-case analyses results and this may help in comparison of the results under specific cost-effectiveness thresholds set from different perspectives. Additionally, to compare cost-effectiveness estimates in cost per QALY, all ICER estimates were adjusted to US$ 2020 by using PPP and inflation rate because costs can be significantly underestimated if not appropriately inflated [30, 34]. Several studies have suggested that G-CSF prophylaxis strategy following chemotherapy for all NHL patients at high risk for FN (> 20%) decreases morbidity and mortality, and our systematic review underpins these recommendations [4, 18, 32, 34]. However, most of the reviewed studies were from high-income countries, and therefore we recommend similar studies to be conducted in low-and middle- income countries.

This review has some limitations. First, the types of economic models used in reviewed studies varied in their model structure, time horizon, perspectives, health outcome measures, and assumptions which limited us from providing a definitive conclusion. Second, this review was limited to journal articles published in English and might miss articles published in other languages. Despite these limitations, our systematic review provides a summary of the cost-effectiveness of pegfilgrastim versus filgrastim for primary and secondary prophylaxis for chemotherapy-induced FN and thus can inform policy decisions regarding clinical care and resource allocation of appropriate interventions for chemotherapy-induced FN management.

## Conclusions

Most studies showed that pegfilgrastim is cost-effective compared to filgrastim as primary and secondary prophylaxis for chemotherapy-induced FN among patients with lymphoma at a cost-effectiveness threshold of US$ 50,000 per QALY gained. Future cost-effectiveness studies regarding G-CSFs should pay attention to influential parameters presented in this review, such as medication cost, FN relative risk, case-fatality rate, length of hospital stay, and baseline FN risk, and pegfilgrastim mortality benefit. We further recommend that future economic evaluations of pegfilgrastim be undertaken from a societal viewpoint.

## Supplementary Information

**Additional file 1: **Search strategy.

**Additional file 2: Supplementary file 2**. JBI critical appraisal checklist for economic evaluations for quality assessment of the included studies.

## Data Availability

All the materials are uploaded as supplementary files.

## Abbreviations

G-CSF: Granulocyte colony-stimulating factors
FN: Febrile neutropenia
ICER: Incremental cost-effectiveness ratio
LYs: Life-years
NHL: Non-Hodgkin Lymphoma
R-CHOP: Cyclophosphamide, doxorubicin, vincristine, and prednisone with or without rituximab
QALY: Quality-adjusted life-year
