# Supplementary material for: Cost-effectiveness of pegfilgrastim versus filgrastim for prevention of chemotherapy-induced febrile neutropenia in patients with lymphoma: a systematic review
Source: BMC Health Serv Res. 2022 Dec 30;22:1600. doi: 10.1186/s12913-022-08933-z (PMC9805270; doi:10.1186/s12913-022-08933-z)
Supplement: Supplementary file 1 — Additional file 1: Search strategy. [file 12913_2022_8933_MOESM1_ESM.docx]

**Search strategy**

- PubMed/Medline
- EMBASE
- Cochrane library

|  | **PubMed/Medline** | **Search results** |
| --- | --- | --- |
| #1 | "Lymphoma"[Mesh] OR "Hodgkin lymphoma"[tw] OR "Non-Hodgkin lymphoma "[tw] OR "Lymphoma, Non-Hodgkin"[tw] OR "Burkett’s lymphoma"[tw] OR "B-cell lymphoma"[tw] OR "Diffuse large B-cell Lymphoma"[tw] OR "T-cell lymphoma"[tw] OR "Follicular lymphoma"[tw] OR "Mantle cell lymphoma"[tw] OR "Primary mediastinal B-cell lymphoma"[tw] OR "Small lymphocyte lymphoma"[tw] | 208,221 |
| #2 | "Febrile neutropenia"[Mesh] OR “Neutropenia, febrile” OR Neutropenia [tw] OR "Neutropenic fever"[tw] OR "Chemotherapy-induced febrile neutropenia"[tw] OR "Primary prophylaxis"[tw] OR "Secondary prophylaxis"[tw] | 49,685 |
| #3 | "Filgrastim "[Mesh] OR "Granulocyte colony-stimulating factor"[tw] OR Neupogen [tw] OR "filgrastim-sndz"[tw] OR "Pegfilgrastim" [Supplementary Concept] OR " Polyethylene glycols"[tw] OR Neulasta [tw] | 81,984 |
| #4 | #1 AND #2 AND #3 | 482 |
| #5 | economic/ or economics, hospital/ or economics, medical/ or economics, nursing/ or economics, pharmaceutical/ or models, economic/ or health care sector/ | 1,157,636 |
| #6 | resource allocation/ or "cost allocation"/ or "costs and cost analysis"/ or cost benefit analysis/ or "cost control"/ or "cost savings"/ or "cost of illness"/ or "cost sharing"/ or health care costs/health expenditures/ or capital expenditures/ | 14,358 |
| #7 | "deductibles and coinsurance"/ or Medical savings accounts/ or Direct service costs/ or Drug costs/ or Employer health costs/ or Hospital costs/ or Value of life/ or hospital charges/ or fees, medical/ or exp "fees and charges"/ or exp budgets/ | 263 |
| #8 | ((low adj2 cost*) or (high adj2 cost*) or (health?care adj2 cost*) or (fiscal or funding or financial or finance) or (cost adj2 estimate*) or (cost* adj2 variable) or (unit adj2 cost*) or cost* or (economic* or incremental* or cost*)).tw | 10,551 |
| #9 | (Economic, pharmacoeconomic, price, or pricing).tw. | 1,485 |
| #10 | exp models, economic/ | 326 |
| #11 | (economic model*).tw | 1,170 |
| #12 | markov chains/ | 16,439 |
| #13 | Markov/.tw | 153 |
| #14 | Monte carlo method/ | 41,794 |
| #15 | Monte carlo/.tw | 271 |
| #16 | exp Decision Theory/ | 470 |
| #17 | "Cost-benefit analysis"[Mesh] OR "Cost-effectiveness"[tw] OR "Cost-effectiveness analysis"[tw] OR "Pharmacoeconomic evaluation"[tw] OR "cost-utility analysis"[tw] OR "Economic evaluation"[tw] OR "Cost-Benefit Analysis"[tw] OR "Cost-efficiency"[tw] OR "Cost-utility analyses" [tw] OR "Cost-effectiveness analyses"[tw] | 134,190 |
| #18 | "models, economic"[MeSH Terms] OR ("models"[All Fields] AND "economic"[All Fields]) OR "economic models"[All Fields] OR ("models"[All Fields] AND "economic"[All Fields]) OR "models, economic"[All Fields] | 53,898 |
| #19 | OR/5-18 | 1,157,636 |
| #20 | #4 AND #18 | 66 |
| #21 | Limit 20 to year=”1990-current” | 66 |
|  | **EMBASE** |  |
| #1 | ("Lymphoma"/exp OR "Hodgkin lymphoma" OR "Non-Hodgkin lymphoma" OR Non-Hodgkin" OR "Burkett’s lymphoma" OR "B-cell lymphoma" OR "Diffuse large B-cell Lymphoma" OR "T-cell lymphoma" OR "Follicular lymphoma" OR "Mantle cell lymphoma" OR "Primary mediastinal B-cell lymphoma" OR "Small lymphocyte lymphoma").tw.kw. | 370,790 |
| #2 | (Febrile neutropenia"/exp "Neutropenic fever"/exp OR "Chemotherapy-induced febrile neutropenia" OR Neutropenia* OR Leukopen*). tw,kw. | 249,864 |
| #3 | **("**Filgrastim"/exp OR "Granulocyte colony-stimulating factor" OR Colony-Stimulating factors” OR G-CSF* OR “Neupogen”).tw.kw. | 75,362 |
| #4 | (“Pegfilgrastim”/exp OR "Polyethylene glycol-conjugated Filgrastim" OR “Neulasta”).tw.kw. | 396 |
| #5 | ("Cost-effectiveness"/exp OR “Cost-benefit analysis" OR "Cost-effectiveness analysis" OR "Pharmacoeconomic evaluation" OR "cost-utility analysis" OR "Economic evaluation" OR "Cost-efficiency" OR "Cost-utility analyses" OR “Cost* analysis” OR “Economics”).tw.kw. | 2,229,190 |
| #6 | **#1 AND #2 AND #3 AND #4 AND #5** | 107 |
|  | **Cochrane Library** | |
| #1 | ("Lymphoma" or "Hodgkin's Lymphoma" "Non- Hodgkin's Lymphoma " "Lymphoma, Non-Hodgkin's" or "Burkett’s lymphoma" or "B-cell lymphoma" "Diffuse large B-cell Lymphoma" or "T-cell lymphoma" or "Follicular lymphoma" or "Mantle cell lymphoma" or "Primary mediastinal B-cell lymphoma" or "Small lymphocyte lymphoma")ti, ab, kw | 103 |
| #2 | ("Febrile neutropenia" or Neutropenia or "Neutropenic fever" or "Chemotherapy-induced febrile neutropenia" or "Primary prophylaxis" or "Secondary prophylaxis") ti, ab,kw | 53 |
| #3 | (Filgrastim or "Granulocyte colony-stimulating factor" or Neupogen)ti,ab,kw | 11 |
| #4 | (Pegfilgrastim or " Polyethylene glycol-conjugated filgrastim" or Neulasta):ti,ab,kw | 628 |
| #5 | ("Cost-benefit analysis" or "Cost-effectiveness" or "Cost-effectiveness analysis" "Pharmacoeconomic evaluation" "cost-utility analysis" or "Economic evaluation" or "Cost-Benefit Analysis" or Cost-efficiency or "Cost-utility analyses" or "Cost-effectiveness analyses") ti, ab,kw | 462 |
| #6 | #1 OR #2 OR #3 OR #4 | 779 |
| #7 | #5 AND #6 | **27** |
