# Supplementary material for: Cost-effectiveness of pegfilgrastim versus filgrastim for prevention of chemotherapy-induced febrile neutropenia in patients with lymphoma: a systematic review
Source: BMC Health Serv Res. 2022 Dec 30;22:1600. doi: 10.1186/s12913-022-08933-z (PMC9805270; doi:10.1186/s12913-022-08933-z)
Supplement: Supplementary file 2 — Additional file 2: Supplementary file 2. JBI critical appraisal checklist for economic evaluations for quality assessment of the included studies. [file 12913_2022_8933_MOESM2_ESM.docx]

**Supplementary file 2: JBI critical appraisal checklist for economic evaluations for quality assessment of the included studies**

| No. | Checklist question | Fust *et al.* 2017 [23] | Ravangard *et al.*2017 [34] | Wang *et al.* 2016 [24] | Lathia *et al,* 2013 [19] | Perrier *et al*.2013 [33] | Sebban *et al.*2012 [32] | Whyte *et al*. 2011 [21] | Lyman *et al.* 2009 [22] |
| --- | --- | --- | --- | --- | --- | --- | --- | --- | --- |
| 1. | Is there a well-defined question? (10%) | Y | Y | Y | Y | Y | Y | Y | Y |
| 2. | Is there comprehensive description of alternatives? (10%) | Y | Y | Y | Y | Y | Y | Y | Y |
| 3. | Are all important and relevant costs and outcomes for each alternative identified? (10%) | Y | Y | Y | Y | Unclear | Unclear | Y | Y |
| 4. | Has clinical effectiveness been established? (10%) | Y | Y | Y | Y | N | N | Y | Y |
| 5. | Are costs and outcomes measured accurately? (5%) | Y | Y | N | Y | Y | Y | Y | Y |
| 6. | Are costs and outcomes valued credibly? (5%) | Y | Y | N | Y | Y | Y | Y | Y |
| 7. | Are costs and outcomes adjusted for differential timing? (10%) | Y | Y | NA | NA | NA | NA | Y | Y |
| 8. | Is there an incremental analysis of costs and consequences? (10%) | Y | Y | Y | Y | Y | Y | Y | Y |
| 9. | Were sensitivity analyses conducted to investigate uncertainty in estimates of cost or consequences? (10%) | Y | Y | Y | Y | Y |  | Y | Y |
| 10. | Do study results include all issues of concern to users? (10%) | Y | N | Y | Y | N |  | Y | Y |
| 11. | Are the results generalizable to the setting of interest in the review? (10%) | Y | N | Y | Y | Y |  | Y | Y |
| 12. | Overall appraisal: Include/Exclude/Seek further info, no relevant =NR, Unclear/not applicable | I | I | I | I | I | I | I | I |

***Y:*** *Yes;* ***N:*** *No;* ***NA:*** *Not applicable;* ***I:*** *Include in the review*
